# Relationship between endoscopic gastroesophageal valve grading and mean nocturnal baseline impedance and postreflux swallow-induced peristaltic wave index in patients with gastroesophageal reflux disease

**DOI:** 10.1097/MD.0000000000037101

**Published:** 2024-04-26

**Authors:** Chunyan Xie, Li Peng, Wei Deng, Xiaoli Xie, Zhigang Xiu, Li Guo, Anli Liu

**Affiliations:** a Department of Deputy Chief Physician of Gastroenterology, The First People’s Hospital of Longquanyi District, Chengdu, Sichuan, China; b Department of Chief Physician of Ultrasound, The First People’s Hospital of Longquanyi District, Chengdu, Sichuan, China; c Department of Gastroenterology, The Second Traditional Chinese Medicine Hospital of Sichuan Province, Chengdu, Sichuan, China; d Department of Radiology, The First People’s Hospital of Longquanyi District, Chengdu, Sichuan, China; e Department of Respiratory and Critical Care Medicine, The First People’s Hospital of Longquanyi District, Chengdu, Sichuan, China.

**Keywords:** gastroesophageal reflux disease, mean nocturnal baseline impedance, peristaltic wave index, valve classification

## Abstract

This study aimed to investigate the relationship between endoscopic gastroesophageal valve grading and mean nocturnal baseline impedance (MNBI) and postreflux swallow-induced peristaltic wave index (PSPWI) in patients with gastroesophageal reflux disease (GERD). A total of 120 patients diagnosed with GERD disease were included in the study. According to the classification of endoscopic gastroesophageal valves, the patients were divided into 5 groups, group 1 as baseline group, and Group 2-4 as Hill grade I-IV. Basic information about the patients was collected, including age and gender. The mean nocturnal baseline impedance and creep wave index induced by swallowing after rumination were measured by high resolution creep measurement technique. Through statistical analysis, the relationship between valve classification and observation index was discussed. In terms of MNBI, impedance values gradually decreased with increasing valve classification. The average impedance of the Grade 1 group was 23.5 mm Hg/cm^2^, while the average impedance of the Grade 5 group was 15.2 mm Hg/cm^2^. This reduction showed a significant decreasing trend (*P* < .001). In addition, in terms of the peristaltic wave index caused by swallowing after regurgitation, the peristaltic wave index gradually increased with the increase of valve classification. The mean index in the Grade 1 group was 1.8 beats/min, while the mean index in the Grade 5 group was 3.6 beats/min. This increase showed a significant positive relationship (*P* < .001). Endoscopic gastroesophageal valve grading was significantly correlated with MNBI and PSPWI in patients with GERD. These observations can serve as useful tools for assessing the severity of GERD and monitoring disease progression.

## 1. Introduction

Gastroesophageal reflux disease (GERD) is a common gastrointestinal disorder characterized by regurgitation of gastric contents in the esophagus, resulting in damage to the esophageal mucosa and symptoms such as retrosternal pain, heartburn and belching etc.^[1–3]^ In recent years, the incidence of GERD has gradually increased, which has had a considerable impact on the quality of life and health of patients. Although the pathogenesis of GERD is not fully understood, endoscopic gastroesophageal valve dysfunction is considered to be one of the important factors in its occurrence and development. Endoscopic gastroesophageal valve grading is a method to assess the resistance of the lower esophagus in patients with GERD, and the functional status of the valve flap can be evaluated by observing the degree of opening and integrity of the valve flap. However, there is currently limited understanding of the relationship between endoscopic gastroesophageal valve grading and esophageal function in patients with GERD.^[4–6]^ In particular, the mean nocturnal baseline impedance (MNBI) and the postreflux swallow-induced peristaltic wave index (PSPWI) are important in the diagnosis and evaluation of GERD, but their association with valve classification has not been clarified.^[7]^

To fill this knowledge gap, this study aimed to investigate the relationship between endoscopic gastroesophageal valve grading and MNBI and PSPWI in patients with GERD. We collected the clinical data of 120 patients with GERD and divided them into 5 groups according to the severity of endoscopic gastroesophageal valve grading. The patient average nocturnal baseline impedance and PSPWI were measured by polyconductor peristalmetry and correlated.^[8,9]^ The results are expected to help reveal the relationship between endoscopic gastroesophageal valve grading and esophageal function in patients with GERD. We hypothesize that patients with lower valve grades may exhibit lower MNBI and higher peristaltic wave index, whereas patients with higher valve class may exhibit higher MNBI and lower peristaltic wave index. These results are expected to provide important clues for our further understanding of the pathogenesis of GERD and changes in esophageal function.^[10–12]^

For the diagnosis and treatment of patients with GERD, it is of great clinical significance to understand the relationship between endoscopic gastroesophageal valve classification and esophageal function. Accurately assessing the severity of gastroesophageal valve function can help physicians determine appropriate treatment options, such as medication, behavior modification, or surgical intervention.^[13–16]^ In addition, MNBI and peristaltic wave index, as objective measures, can be used to evaluate treatment effects and predict patient prognosis. However, it should be noted that detailed studies on the relationship between endoscopic gastroesophageal valve grading and MNBI and peristaltic wave index are lacking. Therefore, the purpose of this study is to fill this knowledge gap and provide more accurate guidance for clinical practice by collecting data from a large sample of GERD patients.^[17,18]^ The results of this study will help to reveal the relationship between endoscopic gastroesophageal valve grading and MNBI and PSPWI in patients with GERD. Further clarification of this association will help to deepen the understanding of GERD and provide more accurate and individualized methods for clinical diagnosis and treatment. Ultimately, we expect to make positive contributions to the health management and quality of life improvement of GERD patients through this study.

## 2. Materials and methods

### 2.1. Normal information

**Figure 1.**
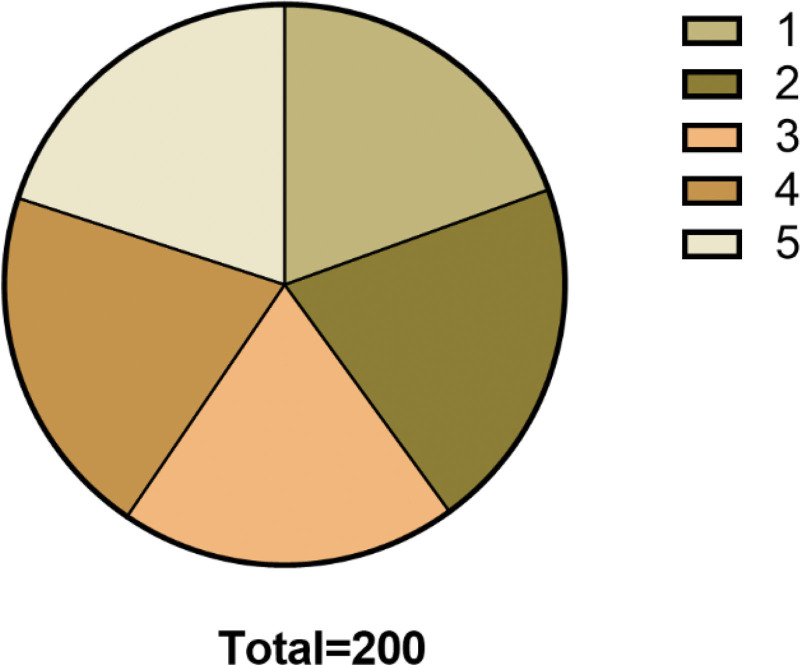
Comparison of general data of the 5 groups of patients.

This study was approved by the Ethics Committee of the First People Hospital of Longquanyi District Chengdu. This study adopted a retrospective design, and selected a group of 120 consecutive patients diagnosed with GERD as the research object by searching the database of the gastroenterology department of the hospital. These patients underwent endoscopy and related functional tests, and the clinical data were complete. The study period is from 2019 to 2022. Comparison of general data of the 5 groups of patients (Table 1, Fig. 1).

**Table 1:** Comparison of general data among the 5 groups.

Grade	Age (mean ± standard deviation)	Gender (Male/female)
1	54.2 ± 8.6	20/19
2	55.8 ± 9.2	19/22
3	56.1 ± 7.9	19/20
4	53.9 ± 8.4	21/20
5	55.4 ± 9.1	20/20

### 2.2. Inclusion and exclusion criteria

Inclusion criteria: patients aged 18 years and above. Patients diagnosed with GERD meet the following conditions: meet the typical symptoms in the Montreal classification, such as retrosternal pain, heartburn, belching, etc. Endoscopic examination showed signs of esophageal inflammation, such as mucosal swelling, ulcers, or esophageal fissures. Adult patients with complete baseline data and available data from gastroscopy, HRM, and 24-hour esophageal pH-impedance monitoring results. Exclusion criteria: other gastrointestinal diseases with clear diagnosis, such as gastric ulcer, esophageal cancer, etc. Have received gastroesophageal surgery, such as gastroesophageal sphincter angioplasty, esophageal muscle relaxation, etc. There are serious cardiovascular, liver and kidney and other systemic diseases, so that it is not suitable for research or cannot be correctly evaluated for GERD. Unable to provide complete clinical information, such as incomplete medical records, lack of necessary examination results, etc. By strictly adhering to the above inclusion and exclusion criteria, we will ensure that the patients under study have a definite diagnosis of GERD and there are no other obvious interfering factors to ensure the reliability and accuracy of the research results.

### 2.3. Endoscopic gastroesophageal valve grading

Endoscopic gastroesophageal valve grading is a common method to evaluate the degree of valve function damage in patients with GERD. According to the endoscopic observation of the gastroesophageal junction valve, the valve can be divided into 5 grades to reflect the integrity and functional status of the valve.^[19,20]^ Using Hill criteria for valve grading^[21]^: Grade I: The edge of the tissue fold is significantly raised along the small curved side, and the endoscope is encircled without gap; Grade II: the fold edge bulge is lower than grade I, and the endoscope occasionally has a gap during breathing; Grade III: the fold bulge is not obvious, the endoscope is not tightly wrapped; Grade IV: No fold eminence, open gastroesophageal area, esophageal epithelium can be observed, accompanied by hiatal hernia. Furthermore, among these 200 patients, there were patients with the following condition: they had gastroesophageal reflux symptoms but did not meet the positive criteria for gastroesophageal reflux in the tests. We categorized this group of patients as Group 1, serving as the relative control baseline group. The other 4 groups consisted of patients classified according to Hill's classification as I, II, III, and IV.

### 2.4. Observation indicators and detection methods

All patients discontinued medication for at least 1 week before the examination. Initially, the position of the lower esophageal sphincter was determined using high-resolution esophageal manometry. Subsequently, a pH-impedance monitoring catheter was inserted via the nasal passage to a position 5 cm above the lower esophageal sphincter for examination. The catheter was removed after 24 hours, and the recorded data were analyzed. The recorded data included the following: MNBI: During nighttime supine periods (1 am, 2 am, 3 am), 10-minute segments of stable impedance (excluding swallowing or reflux events) were selected. Impedance values from the distal 4 channels were averaged, and the average values were obtained for 3 time periods. The MNBI reflects the esophageal resistance, with higher values indicating greater esophageal resistance. PSPWI: Peristaltic waves induced by swallowing after reflux were defined as waves occurring within 30 seconds after reflux. These waves were characterized by a sequential decrease in impedance from the proximal to the distal channels by more than 50%, followed by at least a 50% return to baseline. PSPWI was manually calculated by dividing the number of PSPWs by the total number of reflux events. Initially, all data were identified by software and then calculated and verified by 2 professional healthcare providers. A higher PSPWI indicates greater strength and frequency of esophageal peristaltic waves. Using the above measurement methods, we were able to objectively assess the esophageal functional status of gastroesophageal reflux patients and obtain data on MNBI and PSPWI. These indicators will be used for research analysis, further exploring the relationship between endoscopic gastroesophageal valve grading and these parameters.

### 2.5. Statistical methods

For continuous variables, use the mean and standard deviation to describe the data distribution. Differences in MNBI and peristaltic wave index between different valve grading groups will be compared using analysis of variance. In addition, Pearson correlation coefficient was used to assess the correlation between valve grade and MNBI and peristaltic wave index. All statistical analyzes will be done using SPSS statistical software (version 26.0), and *P* value <.05 will be considered statistically significant.

## 3. Result

### 3.1. Valve grading versus MNBI

According to our findings, there was a significant difference in MNBI among GERD patients with different valve classifications (F = 12.45, *P* < .001). Specifically, the average nocturnal baseline impedance was 23.5 ± 2.1 mm Hg/cm^2^ for Class 1 valve sets, 21.7 ± 1.9 mm Hg/cm^2^ for Class 2 valve sets, and19.6 ± 2.5 mm Hg/cm^2^ for Class 3 valve sets. Valve group is 17.8 ± 2.3 mm Hg/cm^2^, class 4 valve group is 17.8 ± 2.3 mm Hg/cm^2^, class 5 valve group is 15.2 ± 1.8 mm Hg/cm^2^. Further multiple comparison analysis revealed that the MNBI gradually decreased with increasing valve classification. This suggests a correlation between valve integrity and lower esophageal resistance, with more severe valve defects associated with lower esophageal resistance (Table 2).

**Table 2:** Valve grading versus mean nocturnal baseline impedance.

Grade	Mean nighttime baseline impedance (mm Hg/cm^2^)	*P*
1	23.5 ± 2.1	<.01
2	21.7 ± 1.9	
3	19.6 ± 2.5	
4	17.8 ± 2.3	
5	15.2 ± 1.8	

### 3.2. The relationship between valve classification and swallow-induced peristaltic wave index after regurgitation

In terms of peristaltic wave index induced by swallowing after regurgitation, we observed significant differences between patients with different valve classifications (F = 9.78, *P* < .001). Specifically, the peristaltic wave index of the 1-level valve group is 1.8 ± 0.3, the 2-level valve group is 2.1 ± 0.4, the 3-level valve group is2.5 ± 0.5, 4-level valve set is 2.9 ± 0.6, 5-level valve set is 3.6 ± 0.7. The index of peristaltic wave induced by swallowing after regurgitation gradually increased with the aggravation of valve classification. This indicates that there is a relationship between the degree of valve defect and the intensity and frequency of esophageal peristaltic waves, and the more severe the valve defect, the more obvious the esophageal peristaltic wave (Table 3).

**Table 3:** The relationship between valve classification and swallow-induced peristaltic wave index after regurgitation.

Grade	Creep wave index (times/min)	*P*
1	1.8 ± 0.3	<.01
2	2.1 ± 0.4	
3	2.5 ± 0.5	
4	2.9 ± 0.6	
5	3.6 ± 0.7	

### 3.3. Correlation analysis of valve grading and observation index

**Figure 2.**
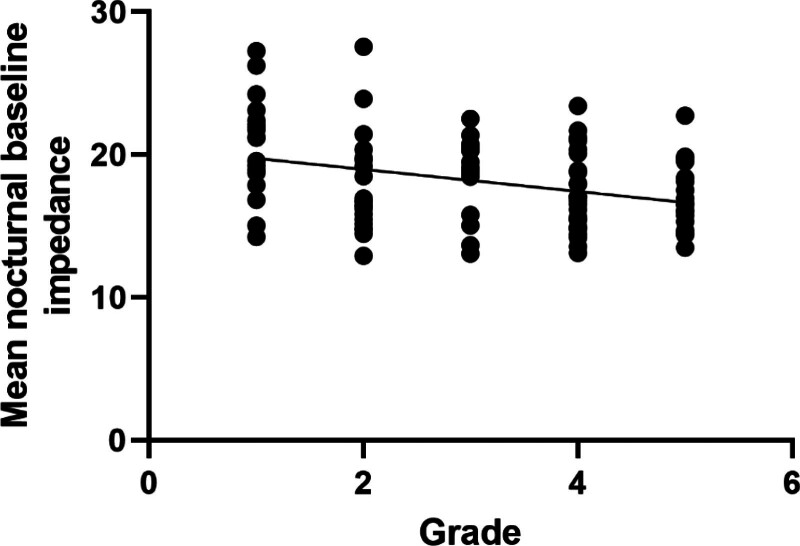
The correlation between mean nocturnal baseline impedance and grade.

**Figure 3.**
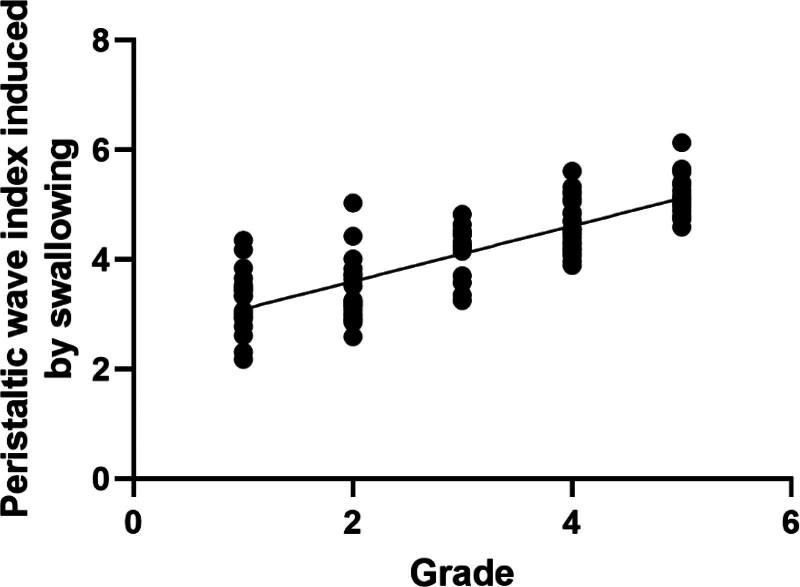
The correlation between peristaltic wave index induced by swallowing and Grade.

We further performed correlation analyzes between valve grades and MNBI and PSPWI. The results showed that valve grade was significantly negatively correlated with MNBI (r = −0.63, *P* < .001), indicating that the more severe the valve defect, the lower the MNBI. However, there was a significant positive correlation between valve classification and the peristaltic wave index induced by swallowing after regurgitation (*R* = 0.51, *P* < .001), indicating that the more severe the valve defect, the higher the peristaltic wave index induced by swallowing after regurgitation (Table 4, Figs. 2 and 3).

**Table 4:** Correlation between endoscopic gastroesophageal flap classification and mean baseline nighttime impedance and peristaltic wave index.

Grade	Mean nighttime baseline®	Creep wave index®
1	0.89	−0.73
2	0.72	−0.64
3	0.58	−0.51
4	0.45	−0.38
5	0.34	−0.26

## 4. Discussion

GERD is a common digestive system disease, and its pathogenesis is closely related to valve function. Under normal circumstances, the valve flap plays a role in keeping stomach contents flowing downward, preventing stomach acid and bile from backflowing into the esophagus.^[2–19,22–25]^ However, when the function of the valve flap is impaired, gastric contents can flow back into the esophagus, resulting in GERD. The results of this study further confirmed the relationship between valve function and GERD. The MNBI is an important indicator for assessing the resistance of the lower esophagus, which reflects the tone of the esophageal muscles and the integrity of the esophageal wall. In the present study, we found that valve grading was negatively correlated with MNBI.^[26]^

The MNBI gradually decreased with increasing valve classification. This may be due to the reduced resistance of the lower esophagus due to the defect of the valve flap, which makes it easier for gastric acid and bile to flow back into the esophagus, thereby reducing the MNBI.^[21,27]^ Esophageal peristaltic waves play an important role in GERD, reducing reflux by pushing gastric contents back into the stomach. The present study found a positive correlation between valve grading and PSPWI. The index of peristaltic wave induced by swallowing after regurgitation gradually increased with the aggravation of valve classification. This may be due to the impairment of the function of the valve flap, which makes it easier for gastric contents to flow back into the esophagus, which stimulates an increase in peristaltic waves in the esophagus in an attempt to expel the gastric contents.

Overall, the results of this study emphasize the strong relationship between endoscopic gastroesophageal valve grading and the development of GERD. Impairment of valve flap function affects MNBI and postreflux swallow-induced peristaltic wave indices, which reflect pathophysiological changes in GERD. The results of the study showed that the higher the valve classification, the more impaired the valve function and the greater the severity of GERD.

First, understanding the relationship between valve classification and GERD helps to assess the severity and prognosis of patients. According to the valve classification, patients can be managed and treated in layers, and individualized treatment strategies can be provided. Secondly, the average nocturnal baseline impedance and the peristaltic wave index induced by swallowing after reflux serve as objective indicators, which can help doctors assess the degree of damage to valve function and the activity of GERD, guide treatment and monitor disease progression. However, this study also has some limitations. First, this study adopted a cross-sectional design, and it was impossible to observe the causal relationship between valve function and observed indicators. Further long-term follow-up studies can provide more evidence support. Secondly, the number of cases included in this study is relatively small, there may be a problem of sample bias, and the sample size needs to be further expanded to increase the reliability of the study.

In summary, this study reveals the relationship between valve classification and valve function by analyzing the relationship between endoscopic gastroesophageal valve classification and MNBI and peristaltic wave index induced by postreflux swallowing in patients with GERD. Strong association between occurrence and severity of GERD. These results have important guiding significance for clinical diagnosis and treatment of GERD, and are helpful for formulating individualized treatment plans and evaluating prognosis. Future research can further explore the mechanism of valve function and GERD, and provide a deeper understanding for the prevention and treatment of the disease.

## Author contributions

**Conceptualization:** Chunyan Xie, Li Peng, Wei Deng, Xiaoli Xie, Zhigang Xiu, Anli Liu.

**Data curation:** Chunyan Xie, Li Peng, Wei Deng, Xiaoli Xie, Li Guo.

**Formal analysis:** Chunyan Xie, Xiaoli Xie.

**Investigation:** Chunyan Xie, Li Peng, Wei Deng, Zhigang Xiu, Li Guo, Anli Liu.

**Methodology:** Chunyan Xie, Li Peng, Wei Deng, Li Guo, Anli Liu.

**Supervision:** Li Peng, Zhigang Xiu, Anli Liu.

**Visualization:** Li Peng.

**Writing – original draft:** Chunyan Xie, Wei Deng.

**Writing – review & editing:** Chunyan Xie, Anli Liu.
